# Draft Genome Sequences of Three Porcine Streptococcus suis Isolates Which Differ in Their Susceptibility to Penicillin

**DOI:** 10.1128/MRA.01711-18

**Published:** 2019-02-28

**Authors:** Lisa Niemann, Inga Eichhorn, Petra Müller, Jasmin Brauns, Rolf Nathaus, Franziska Schäkel, Doris Höltig, Michael Wendt, Kristina Kadlec, Stefan Schwarz

**Affiliations:** a Institute of Microbiology and Epizootics, Freie Universität Berlin, Berlin, Germany; b Department of Pharmacology, Toxicology and Pharmacy, University of Veterinary Medicine Hannover, Foundation, Hannover, Germany; c Clinic for Swine, Small Ruminants, Forensic Medicine and Ambulatory Services, University of Veterinary Medicine Hannover, Foundation, Hannover, Germany; d Vet-Team Reken, Reken, Germany; e Institute for Biometry, Epidemiology and Information Processing, WHO Collaborating Centre for Research and Training for Health at the Human-Animal-Environment Interface, University of Veterinary Medicine Hannover, Foundation, Hannover, Germany; f Dairy Herd Consulting and Research Company (MBFG), Wunstorf, Germany; University of Arizona

## Abstract

The draft genome sequences of three Streptococcus suis isolates, IMT40343, IMT40201, and IMT40738, are presented here. These isolates were obtained from bronchoalveolar lavage fluid of healthy and diseased weaners from different German piglet-producing farms and differed in their susceptibility to penicillin.

## ANNOUNCEMENT

Streptococcus suis strains are usually commensals colonizing the respiratory tract of pigs (1). However, they can cause meningitis, pneumonia, septicemia, arthritis, endocarditis, or abortion in pigs (1). Moreover, S. suis has been reported as a zoonotic pathogen, primarily in humans with occupational contact with pigs (2–4). To treat S. suis infections in pigs and humans, β-lactams are recommended. Unfortunately, reduced susceptibility of isolates against β-lactams, especially penicillins, has been reported during the last three decades (5–8).

The S. suis isolates IMT40343, IMT40201, and IMT40738 were obtained from bronchoalveolar lavage fluid of weaners originating from three different German piglet-producing farms. IMT40343 was isolated from a healthy weaner, whereas IMT40738 was from a weaner with respiratory tract disease. IMT40201 originated from a weaner that was cured of a respiratory tract infection and was isolated immediately after treatment with doxycycline. Aliquots of 100 µl of the bronchoalveolar lavage fluid were plated onto Columbia sheep blood agar and incubated for 20 to 24 h at 37°C under aerobic and microaerophilic conditions. Colonies suspected to be S. suis were confirmed by a species-specific PCR and matrix-assisted laser desorption–ionization time-of-flight mass spectrometry (MALDI-TOF MS) as described earlier (9). MIC determination, according to CLSI standards, identified IMT40343 as penicillin susceptible (MIC, ≤0.015 mg/liter), IMT40201 as penicillin intermediate (0.5 mg/liter), and IMT40738 as penicillin resistant (2 mg/liter) (10). The whole-genome sequences (WGSs) of IMT40343, IMT40201, and IMT40738 were determined and analyzed for point mutations in the genes for penicillin-binding proteins (PBPs) that might account for elevated penicillin MICs. The three S. suis isolates were grown for 20 to 24 h at 37°C in brain heart infusion broth under microaerophilic conditions prior to DNA extraction with the QIAamp DNA mini kit (Qiagen, Hilden, Germany). The libraries were prepared using a Nextera XT library preparation kit (Illumina, Inc., USA) according to the manufacturer’s recommendations. The 2 × 300-bp paired-end sequencing in 30-fold multiplexes was performed on the MiSeq (Illumina, Inc.) platform. Genome sequences were *de novo* assembled using the Mimicking Intelligent Read Assembler (MIRA) v4.0 (Biomatters Ltd., New Zealand) with default settings as a plugin within Geneious v10.1.3 (Biomatters Ltd.) (Table 1). The coverage of the sequences was approximately 50-fold.

**TABLE 1:** Characteristics of the draft genome sequences of the three S. suis isolates

Strain (IMT no.)	NCBI reference sequence no.	BioSample no.	Genome size (bp)	No. of contigs	*N*_50_ length (bp)
IMT40201	NZ_RRZQ00000000	SAMN10440285	2,344,245	66	179,911
IMT40738	NZ_RRZO00000000	SAMN10440287	2,458,255	192	34,794
IMT40343	NZ_RRZP00000000	SAMN10440286	2,254,559	42	155,466

The total genome size of IMT40343 was 2,254,559 bp, and that of IMT40201 was 2,344,245 bp, while IMT40738 had a size of 2,458,255 bp. The G+C contents ranged from 41.0 to 41.5%. Gene annotation was performed via the Rapid Annotations using Subsystems Technology (RAST) server (11). For IMT40343 and IMT40201, RAST identified 2,316 and 2,338 coding DNA sequences, with 10 and 13 rRNAs and 48 and 51 tRNAs, respectively. For IMT40738, RAST annotated 2,536 coding sequences with 8 rRNAs and 54 tRNAs. The Geneious BLAST tool identified class A (PBP2x and PBP2b) and class B (PBP1a, PBP2a, and PBP1b) PBPs with identity thresholds between 94 and 100% (12). Analyses of the PBPs revealed an accumulation of amino acid exchanges in PBP2x and PBP2b of IMT40738, followed by that in IMT40201. However, no amino acid alterations within the conserved motifs were detected in all three sequences. Based on the detection of the respective loci in the draft genome sequences, isolates IMT40343 and IMT40201 were classified as serotypes 8 and 21, respectively. However, IMT40738 was not serotypeable, as it harbored a novel *cps* locus (13–15). IMT40343 was assigned to sequence type 308 (ST308) using the multilocus sequence typing (MLST) service of the Center for Genomic Epidemiology (16). In contrast, IMT40201 and IMT40738 were assigned to new STs 1097 and 1098, respectively, by the pubMLST service. No acquired antimicrobial resistance genes were found in IMT40343 using ResFinder, while in IMT40201, the resistance genes *mef*(A), *msr*(D), and *tet*(O) were found. In IMT40738, the resistance genes *erm*(B) and *tet*(O/W/32/O) were detected, in addition to *mef*(A) and *msr*(D) (17).

### Data availability.

The WGSs of the isolates IMT40343, IMT40201, and IMT40738 have been deposited with BioProject number PRJNA505967 at DDBJ/ENA/GenBank under the accession numbers RRZP00000000, RRZQ00000000, and RRZO00000000, respectively. The raw data are available as SRA data with the numbers SAMN10440285 (IMT40201), SAMN10440286 (IMT40343), and SAMN10440287 (IMT40738).
